# Apabetalone Downregulates Fibrotic, Inflammatory and Calcific Processes in Renal Mesangial Cells and Patients with Renal Impairment

**DOI:** 10.3390/biomedicines11061663

**Published:** 2023-06-08

**Authors:** Dean Gilham, Sylwia Wasiak, Brooke D. Rakai, Li Fu, Laura M. Tsujikawa, Christopher D. Sarsons, Agostina Carestia, Kenneth Lebioda, Jan O. Johansson, Michael Sweeney, Kamyar Kalantar-Zadeh, Ewelina Kulikowski

**Affiliations:** 1 Resverlogix Corp., 300, 4820 Richard Road SW, Calgary, AB T3E 6L1, Canada; 2 Resverlogix Inc., 535 Mission St, 14th Floor, San Francisco, CA 94105, USA; 3 Harbor-UCLA Medical Center, University of California Los Angeles, 1000 W Carson St, Torrance, CA 90502, USA

**Keywords:** chronic kidney disease (CKD), fibrosis, inflammation, gene expression, epigenetics, BET proteins, apabetalone

## Abstract

Epigenetic mechanisms are implicated in transcriptional programs driving chronic kidney disease (CKD). Apabetalone is an orally available inhibitor of bromodomain and extraterminal (BET) proteins, which are epigenetic readers that modulate gene expression. In the phase 3 BETonMACE trial, apabetalone reduced risk of major adverse cardiac events (MACE) by 50% in the CKD subpopulation, indicating favorable effects along the kidney–heart axis. Activation of human renal mesangial cells (HRMCs) to a contractile phenotype that overproduces extracellular matrix (ECM) and inflammatory cytokines, and promotes calcification, frequently accompanies CKD to drive pathology. Here, we show apabetalone downregulated HRMC activation with TGF-β1 stimulation by suppressing TGF-β1-induced α-smooth muscle actin (α-SMA) expression, α-SMA assembly into stress fibers, enhanced contraction, collagen overproduction, and expression of key drivers of fibrosis, inflammation, or calcification including thrombospondin, fibronectin, periostin, SPARC, interleukin 6, and alkaline phosphatase. Lipopolysaccharide-stimulated expression of inflammatory genes *IL6*, *IL1B*, and *PTGS2* was also suppressed. Transcriptomics confirmed apabetalone affected gene sets of ECM remodeling and integrins. Clinical translation of in vitro results was indicated in CKD patients where a single dose of apabetalone reduced plasma levels of key pro-fibrotic and inflammatory markers, and indicated inhibition of TGF-β1 signaling. While plasma proteins cannot be traced to the kidney alone, anti-fibrotic and anti-inflammatory effects of apabetalone identified in this study are consistent with the observed decrease in cardiovascular risk in CKD patients.

## 1. Introduction

Chronic kidney disease (CKD) is characterized by progressive loss of kidney function, which is an independent risk factor for cardiovascular disease (CVD) [1,2]. Although CKD affects >10% of individuals worldwide, current therapies to slow CKD progression are limited, and thus many patients with CKD advance to end-stage kidney disease with markedly elevated CVD risk. The high rate of renal complications and mortality worldwide emphasize the urgent need for new and effective therapies. CKD is a consequence of different pathophysiologic conditions, such as diabetes, hypertension, acute kidney injury, and autoimmune disorders. Regardless of how CKD is initiated, renal interstitial fibrosis is a common and progressive pathology in CKD, and the extent of fibrosis correlates with renal insufficiency [3]. The glomerulus is the filtration unit in the kidney. Based on the estimated glomerular filtration rate (eGFR), CKD is classified as moderate (eGFR < 60 mL/min/1.73 m^2^) or severe (eGFR < 30 mL/min/1.73 m^2^). Human renal mesangial cells (HRMCs), and the extracellular matrix (ECM) they produce, provide structural support for glomerular capillaries to enhance filtration [4]. However, HRMC activation to fibroblast-like cells that overproduce ECM is critical for initiation and progression of renal fibrosis [5]. The ensuing accumulation of ECM from activated HRMCs physically constrains capillary blood flow and compromises filtration, which contributes to kidney dysfunction, and eventually organ failure. Activated HRMCs also secrete inflammatory cytokines that elicit immune cell infiltration into the mesangium, leading to exaggerated inflammatory responses that accompany and perpetuate fibrosis [6]. Transforming growth factor (TGF)-β1, a pro-fibrotic cytokine, activates HRMCs in vitro and in vivo [7,8,9], and is a driver of diabetic nephropathy in patients [10]. Countering activation of HRMCs and ensuing fibrosis could alleviate CKD pathology; however, therapeutics targeting TGF-β1 or its receptors were not efficacious or had serious side effects [11,12,13]. Processes downstream of TGF-β1 have therapeutic potential to reduce HRMC activation and consequent renal pathology, but, to date, drugs that target and inhibit HRMC activation do not exist.

Recently, epigenetic mechanisms have been implicated in progression of CKD [14]. Apabetalone (RVX-208) is an orally available small molecule inhibitor of bromodomain and extraterminal (BET) proteins, which are epigenetic readers modulating gene expression involved in fibrosis, inflammation, and calcification [15,16,17,18,19]. BET proteins BRD2, BRD3, BRD4, and BRDT govern gene expression through binding acetylation-dependent sites on histones and transcription factors via their tandem bromodomains (BD) 1 and BD2 to bridge chromatin with transcriptional machinery [20]. Inhibitors of BET proteins (BETi) that target both bromodomains with equal affinity (pan-BETi) have been reported to attenuate renal fibrosis and inflammation in pre-clinical models, validating their therapeutic potential [14,21,22,23,24]. However, toxicity associated with pan-BETi limits their clinical application [25,26]. In contrast, apabetalone, which selectively targets BD2 within BET proteins, has an established, favorable safety profile [27,28]. In pre-clinical models, apabetalone countered fibrosis, inflammatory processes, and ectopic calcification [15,16,17,19,29]. In patients with stable cardiovascular disease, apabetalone treatment increased eGFR, indicating improved kidney function [30]. In CKD patients, apabetalone downregulated plasma markers of endothelial dysfunction, vascular inflammation, calcification, and fibrosis, which are processes that promote cardiovascular disease [31]. In the phase 3 BETonMACE trial, which enrolled patients with diabetes mellitus and a recent acute coronary syndrome, apabetalone reduced the risk of major adverse cardiac events (MACE) in the subpopulation with CKD by 50%, i.e., subjects with eGFR < 60 mL/min/1.73 m^2^ Even though apabetalone had no effect on eGFR in this patient population, the reduction in MACE indicates a favorable response along the kidney–heart axis. However, the efficacy of a BD2-selective BETi to regulate HRMC activation associated with renal fibrosis and inflammation remains unknown. 

Here we show that apabetalone inhibits HRMC activation induced by TGF-β1. Apabetalone not only suppressed expression of ECM components and key drivers of fibrosis induced by TGF-β1 stimulation, but also downregulated inflammatory responses stimulated by TGF-β1 or lipopolysaccharide (LPS). We also observed activated HRMCs upregulate tissue non-specific alkaline phosphatase gene expression, which was downregulated by apabetalone. In all studies, the comparator BETi JQ1 or MZ1 had similar activity as apabetalone, confirming on-target effects. Together, our data show that BET proteins are key pharmacological targets for prevention of HRMC activation leading to kidney fibrosis and dysfunction. Further, in a clinical assessment of subjects with renal impairment (CKD stage 4 or 5 not on dialysis) or matched subjects without renal dysfunction, plasma levels of pro-fibrotic and inflammatory markers were reduced as early as twelve hours after a single dose of apabetalone specifically in those with renal impairment. Downregulation of these markers correlated with our in vitro observations. While circulating proteins cannot be traced to the kidney, reduced circulating levels of pro-fibrotic and inflammatory proteins that operate along the kidney–heart axis are consistent with reduced MACE observed in CKD patients receiving apabetalone in a phase 3 trial [32].

## 2. Materials and Methods

Apabetalone and JQ1 were synthesized by NAEJA Pharmaceuticals (Edmonton, AB, Canada) or IRIX Pharmaceuticals (Florence, SC, USA) [33,34]. The proteolysis targeting chimera (PROTAC) MZ1 was purchased from Tocris Bioscience (Bristol, UK). Recombinant, receptor active TGF-β1 was purchased from StemCell Technologies (Vancouver, BC, Canada) or Abcam (Cambridge, UK), a small molecule inhibitor of TGF-β receptors (TGFBRi) was purchased from Abcam (catalog # ab141890), and LPS (E. coli O111:B4) was purchased from Sigma Aldrich (St. Louis, MO, USA).

Cell culture: HRMCs were purchased from Cell Systems (Kirkland, WA, USA) and cultured in mesangial cell complete medium (ScienCell, Carlsbad, CA, USA catalog # 4201 or Cell Systems # 4Z0–500) on plastic coated with Attachment Factor (Cell Systems) and incubated at 37 °C in a humidified atmosphere enriched with 5% CO_2_.

For TGF-β1 stimulation, we followed the protocol published by Liu et al. [35]. Cells were plated in complete media. After 24 h, cells were incubated overnight (16–18 h) in serum-free RPMI media containing penicillin and streptomycin (P/S). Compounds (BETi and TGFBRi) were prepared in dimethyl sulfoxide (DMSO). Cells were pre-treated with compounds for 1 h in RPMI with P/S; the final concentration of DMSO was 0.05%. Following pre-incubation, TGF-β1 was introduced at 2 ng/mL in fresh RPMI + P/S and the same compound treatments. Treatment durations are indicated in figures. For LPS stimulation, we followed the protocol published by Wu et al. [36]. Cells were plated in low-glucose DMEM containing 10% FBS and P/S. After 24 h, cells were pre-treated for 1 h with compounds, followed by LPS containing media and continuing with the same compound treatments.

Real-time PCR: Relative gene expression was determined by real-time PCR as previously described [37]. Briefly, mRNA was isolated using Catcher PLUS kits according to the manufacturer’s instructions (ThermoFisher, Waltham, MA, USA). TaqMan PCR primers were obtained from Applied Biosystems/Life Technologies. Real-time PCR was used to determine the abundance of the transcript of interest relative to the endogenous control cyclophilin in the same sample using the RNA Ultrasense One-step qRT-PCR kit (ThermoFisher). Data were acquired using a ViiA-7 Real-Time PCR apparatus (Applied Biosystems, Waltham, MA, USA). The analysis was performed as 2^^(C_T_ cyclophilin–C_T_ gene of interest)^ and results were normalized to DMSO treated samples. TaqMan assays used in this study are listed in Supplementary Table S1.

Immunofluorescence Microscopy: Microscope coverslips in 12-well plates were coated with Attachment Factor. HRMC were seeded at 200,000 cells per well. After the treatment period, cells were fixed with 100% ice cold methanol for 5–10 min, then washed 3 times with ice cold PBS. Cells were blocked for non-specific binding of antibodies via 20–30 min incubation with 2% BSA in PBS at room temperature. Antibody staining was conducted in 2% BSA/PBS for 1 h. Cells were washed 3 times with cold PBS and mounted on microscope slides with 12 µL of ProLong Diamond Antifade Mountant with DAPI (ThermoFisher catalog # P36962) and sealed with clear nail polish. Images were acquired on a Zeiss Axioskop 2 Plus microscope using Leica Application Suite software version 4.9.0. α-SMA was stained with Alexa Fluor 488 conjugated monoclonal antibody (1A4; ThermoFisher # 53-9760-82) at 1:200 dilution. Quantification of α-SMA fluorescence was performed with ImageJ (version 1.54d) using the Analyze Particles function to identify the area in each field of view with α-SMA fluorescence.

Toxicity: Treatment-induced cell death was assessed using the FITC Annexin V Apoptosis Detection Kit from BD Biosciences (San Jose, CA, USA) according to the supplier’s instructions. Measurements were taken on a BD FACSCelesta flow cytometer (BD Biosciences) and data analyzed with FlowJo software version 10 (BD Biosciences).

Collagen gel contraction: Collagen gel contraction assays were performed essentially as described previously [38]. Briefly, collagen solution was prepared on ice by combining 2.42 mg/mL Cultrex rat collagen I (R&D Systems, Minneapolis, MN, USA, # 3447-020-01), 10× Minimum Essential Medium (MEM), and a solution of 0.05 N NaOH, 2.2% NaHCO_3_, and 200 mM HEPES pH 7.4 in an 8:1:1 ratio. A quantity of 320 μL of the collagen solution was combined with 80 μL of 1× MEM containing 125,000 HRMCs in a pre-chilled 24-well plate. Gels formed over 30 min at 37 °C in a tissue culture incubator. A quantity of 500 μL of 1× MEM was added and incubation continued overnight. Cytokines and treatment compounds were added, gels were released from plastic using a 25-gauge needle, and incubated for an additional 96 h. Gels were imaged using a ChemiDoc XRS (BioRad, Hercules, CA, USA) with a UV convertor screen. Gel area was quantified with QuantityOne software version 4.6.1 (BioRad).

Picrosirius red staining: HRMCs were plated as describe above, then serum starved overnight in RPMI media containing P/S. Treatments were applied in RPMI containing 0.5% FBS and P/S for 5 days with media changes every 2 days. Cells were fixed with 16% formalin for 10 min, then washed 3 times with phosphate buffered saline (PBS). Collagen was stained with picrosirius red solution (Abcam # ab246832) for 60 min, followed by destaining with 0.1 N HCl. Picrosirius red staining was quantified using a ThermoFisher MultiSkan GO spectrophotometer and SkanIt RE software version 3.2 (ThermoFisher).

ELISA: After 48 h of incubation, HRMC media were clarified of debris by 4 min of centrifugation at 10,000× *g* at 4 °C. Levels of protein secreted into media were determined by ELISA according to the manufacturer’s instructions. ELISA kits for fibronectin, thrombospondin-1, periostin, SPARC, and IL-6 were from Abcam catalog # DFBN10, DTSP10, ab213816, ab220654, and ab178013, respectively. Color development was measured on a ThermoFisher MultiSkan GO spectrophotometer and SkanIt RE software version 3.2.

Cell lysates were prepared as previously described [16]. Total cell protein was determined using the DC Protein Assay (BioRad) and bovine serum albumin (BSA) obtained from BioShop (Burlington, ON, Canada) as a standard. Amounts of secreted protein determined by ELISA were normalized to total cell protein.

Alkaline phosphatase (ALP) enzyme activity: Cell-associated ALP activity was measured using a biochemical assay with 4-methylumbelliferyl phosphate (4-MUP; Sigma Aldrich # M8168) as a substrate [39]. Briefly, HRMCs were plated in 96-well black plates with a clear bottom. One day after plating, cells were treated with test compounds for 48 h as described above. Media were removed, cells washed with PBS, and then 50 µL of assay buffer (50 mM Tris, 1 mM MgCl_2_, 125 mM NaCl pH 9.0) containing 50 µM 4-MUP was added to the live cells. Enzymatically active recombinant human TNAP (R&D Systems, Minneapolis, MN, USA, # 2909-AP) was used as a positive control in wells that did not contain cells. A standard curve was generated using 4-methylumbelliferone (4-MU; Sigma Aldrich # M1508, i.e., the product generated by a phosphatase acting on 4-MUP). After an incubation period, fluorescence was measured on a BioTek Synergy H4 plate reader (Agilent Technologies, Santa Clara, CA, USA) using excitation and emission wavelengths of 365 nm and 445 nm.

RNA sequencing (RNA-seq): HRMCs were grown on 6-well plates, with each treatment group prepared in triplicate. Total RNA was isolated using the RNeasy kit (Qiagen, Redwood City, CA, USA). RNA-seq library preparation, sequencing, and mapping was performed by Novogene (Sacramento, CA, USA) using Illumina Platform PE150. Reads were mapped to the human genome sequence GRCh38. Analysis of differential gene expression between treatment groups was performed using the DESeq2 R package version 1.14.1 [40]; genes were considered differentially expressed when padj < 0.05. ClusterProfiler software version 4 [41] was used for analysis of differentially expressed genes (DEGs) in the Reactome Enrichment database.

Proteomic analysis of human plasma: The effect of a single dose of apabetalone on the plasma proteome was evaluated in 8 subjects with stage 4 or 5 CKD who were not on dialysis and a second cohort of 8 subjects matched to the CKD group but without renal impairment. Patients’ characteristics were described by Wasiak et al. [31]. Inclusion criteria were male or female between 18 and 80 years old, stable renal function for at least 3 months prior to screening, provision of written informed consent, and if female (a) provide a negative pregnancy test at screening and a negative urine pregnancy test at Day-1, (b) post-surgical sterilization, or (c) two years postmenopausal. Subjects with renal impairment must be previously diagnosed with end-stage renal disease (eGFR < 30 mL/min/1.73 m^2^), and not on dialysis. Control subjects were matched for age (±10 years), weight (±20%), and gender, but with eGFR ≥ 60 mL/min/1.73 m^2^. CKD patients had comorbidities that commonly accompany renal disease, including diabetes (1 of 8 subjects), and hypertension (5 of 8 subjects). All subjects received a single 100 mg oral dose of apabetalone. Prior to receiving apabetalone, then 6, 12, and 24 h after, plasma was collected for proteomic analysis using the SOMAscan 1.3 k platform (SomaLogic, Boulder, CO, USA). Upstream regulator analysis was performed using QIAGEN’s Ingenuity Pathway Analysis software released July 2021 (IPA; QIAGEN, Redwood City, CA, USA). Informed consent was obtained from all subjects, and the procedures complied with all principles outlined in the Declaration of Helsinki. The complete study protocol, as well as characteristics of patients, have been reported by Wasiak et al. [31].

Statistical Analysis: One-way ANOVA followed by Dunnett’s or Tukey Multiple Comparison Tests, the Benjamini–Hochberg procedure for adjusted *p*-value, or Wilcoxon signed-rank test were applied as indicated in figure legends. Except for RNA-seq, all experiments were performed at least 2 independent times. RNA-seq included 3 independent replicates per treatment group.

## 3. Results

### 3.1. Apabetalone Suppresses TGF-β1-Mediated HRMC Activation

**Figure 1:**
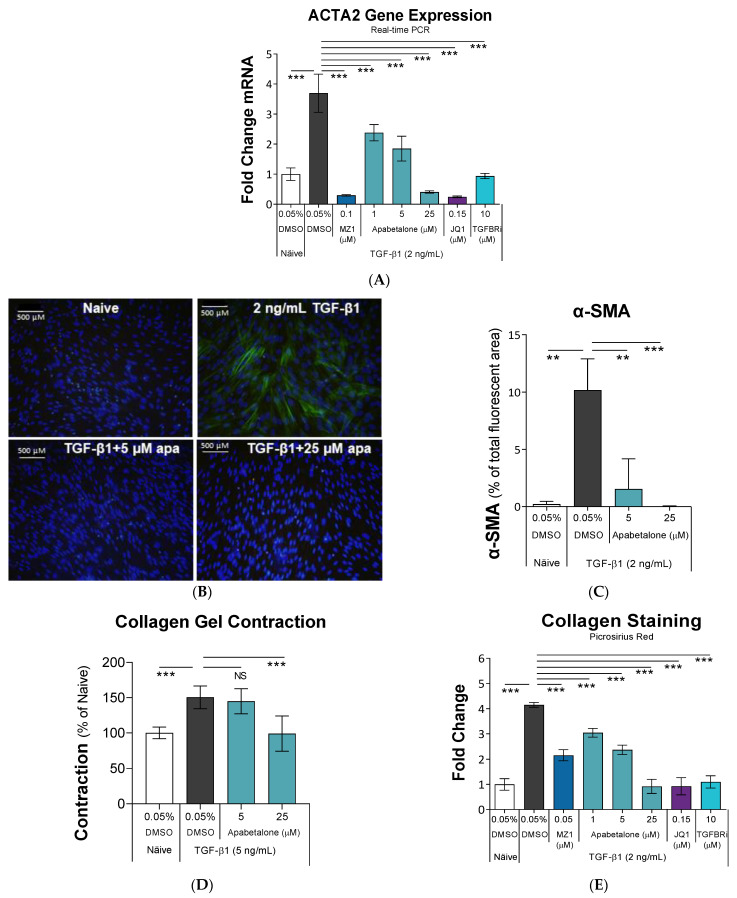
Apabetalone blocks TGF-β1-induced HRMC activation. (**A**): HRMCs were treated with TGF-β1 ± BETi or TGFBRi for 24 h followed by gene expression analysis by real-time PCR (*n* = 5). (**B**): Representative images of HRMCs treated with TGF-β1 ± apabetalone for 48 h, followed by immunofluorescence microscopy for α-SMA (green); nuclei were stained with DAPI (blue); apa = apabetalone. (**C**): Fluorescence intensity of α-SMA was quantified as percent of the image area. (**D**): Collagen gel contraction was evaluated after 4 days of treatment (*n* = 6). (**E**) Collagen deposition was evaluated by picrosirius red staining after 5 days of treatment (*n* = 4). Data in bar graphs are the mean ± SD. Statistical analysis by one-way ANOVA followed by Dunnett’s Multiple Comparison Test. *** *p* < 0.001, ** *p* < 0.01, NS not significant. ACTA2: α-SMA gene. α-SMA: alpha smooth muscle actin. TGF-β1: Transforming growth factor β1. Apabetalone: BD2-selective BET inhibitor. JQ1: pan-BET inhibitor. MZ1: PROTAC that directs BET proteins for degradation. TGFBRi: small molecule inhibitor of the TGF-β receptor.

TGF-β1 treatment activates HRMCs to fibroblast-like cells with a contractile phenotype and increased production of ECM and inflammatory mediators [8]. To understand the effects of BETi on HRMC activation, we treated cells with TGF-β1, with or without BETi. To ensure on-target effects of BETi versus properties unique to apabetalone, BETi with different chemical scaffolds and modes of activity were included. Whereas apabetalone selectively targets BD2 within BET proteins, JQ1 is a pan-BETi with equal affinity for BD1 and BD2 [33], while MZ1 is a PROTAC that directs BET proteins for degradation [42]. A hallmark of pro-fibrotic HRMC activation is elevated expression of α-smooth muscle actin (α-SMA, encoded by the *ACTA2* gene) [43]. As shown in Figure 1A, 24 h of TGF-β1 treatment induced *ACTA2* gene expression by 3.7-fold (white vs. black bars). Apabetalone dose dependently blocked TGF-β1-induced *ACTA2* gene expression. Comparator BETi JQ1 and MZ1 also reduced *ACTA2* mRNA in TGF-β1-stimulated cells. The data indicate BET proteins are involved in TGF-β1-stimulated *ACTA2* gene expression that can be suppressed with BETi. A small molecule inhibitor of the TGF-β receptor (TGFBRi) abolished the response to TGF-β1 treatment as expected (Figure 1A). 

Next, we examined the effects of apabetalone on α-SMA subcellular localization. α-SMA-positive filaments were readily apparent by immunofluorescence microscopy in TGF-β1-treated cells versus naïve (Figure 1B—green stain, with α-SMA fluorescence quantified in Figure 1C), consistent with TGF-β1-mediated induction of the *ACTA2* mRNA (Figure 1A), and indicative of a contractile phenotype. When apabetalone was present during TGF-β1 stimulation, α-SMA filaments were not detected, suggesting HRMC activation was suppressed. This could also arise if apabetalone was toxic to HRMCs. However, staining of DNA with DAPI was consistent between treatment groups, showing no readily apparent effects of apabetalone on the number of nuclei, a proxy for cell number (Figure 1B—blue stain). Compound-induced toxicity was further evaluated using annexin V and propidium iodide staining by flow cytometry, which showed none of the treatments induced cell death (Supplementary Figure S1).

When placed in a collagen gel, HRMCs are able to contract, which is enhanced with TGF-β1 stimulation [44]. We used collagen gel contraction (CGC) assays to assess transition of HRMCs to a contractile phenotype associated with renal pathology. As shown in Figure 1D, TGF-β1 enhanced CGC by >50%, whereas co-incubation with 25 µM apabetalone completely abolished TGF-β1-induced CGC (*p* < 0.001). 

Collagen is the main structural element of the ECM, and is overproduced in activated HRMCs [45]. We applied picrosirius red staining to monitor collagen deposition. On day 5 of TGF-β1 stimulation, collagen staining increased 4.2-fold, but this elevation was countered by apabetalone and other BETi (Figure 1E), indicative of suppressed ECM expansion. For this assay, the concentration of MZ1 was reduced from 0.1 to 0.05 µM to avoid toxicity arising from the extended treatment protocol. The combined results from α-SMA expression, contraction assays, and collagen production show apabetalone suppresses TGF-β1-induced HRMC activation to a pro-fibrotic state.

### 3.2. Apabetalone Blocks TGF-β1 Mediated Expression of Key Drivers of Fibrosis

**Figure 2:**
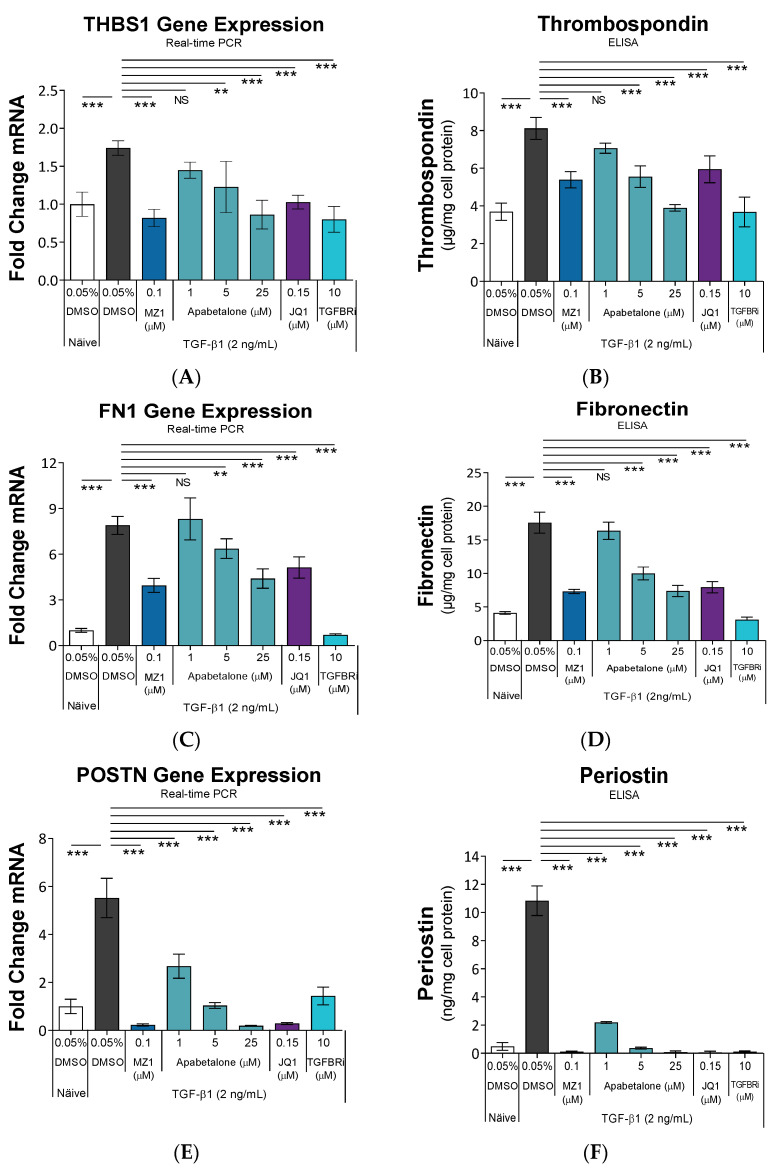

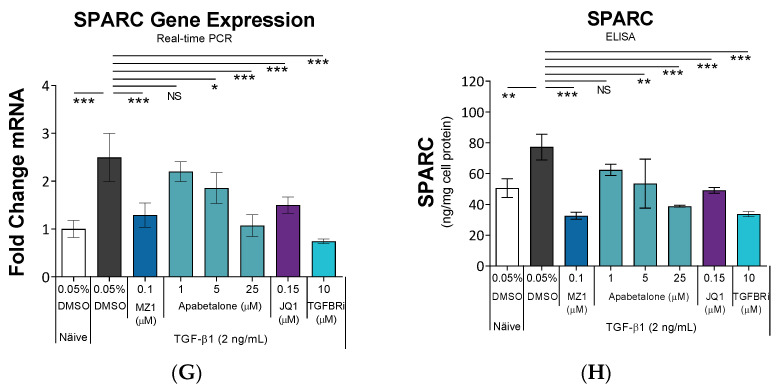
BET proteins regulate expression of key drivers of fibrosis in HRMCs. HRMCs were treated with TGF-β1 ± BETi or TGFBRi for 24 h, followed by gene expression analysis by real-time PCR (left column (**A**,**C**,**E**,**G**); *n* = 4 or 5). For secreted proteins (right column (**B**,**D**,**F**,**H**)), HRMCs were treated for 48 h. Cell culture media were clarified of debris by centrifugation, and the indicated proteins quantified by ELISA (*n* = 3). Data are presented as mean ± SD. Statistical analysis by one-way ANOVA followed by Dunnett’s Multiple Comparison Test. * *p* < 0.05, ** *p* < 0.01, *** *p* < 0.001, NS not significant. *THBS1*: thrombospondin 1 gene. *FN1*: fibronectin gene. *POSTN*: periostin gene. *SPARC* gene and SPARC protein: secreted protein acidic and rich in cysteine. TGF-β1: Transforming growth factor β1. Apabetalone: BD2-selective BET inhibitor. JQ1: pan-BET inhibitor. MZ1: PROTAC that directs BET proteins for degradation.

We expanded our mechanistic assessment to include BETi effects on essential regulators of HRMC activation and ECM expansion. In inflammatory or fibrotic kidney disease, thrombospondin-1 (THBS1, encoded by the *THBS1* gene) is the critical activator of TGF-β1 from its latent form to a receptor active conformation [46,47]. Fibronectin (encoded by the *FN1* gene) is a component of the ECM, and overproduction of fibronectin in the kidney is associated with fibrosis [3]. Periostin (encoded by the *POSTN* gene) is a matricellular protein that promotes inflammation and enhances fibrosis in HRMCs [48]. Secreted protein acidic and rich in cysteine (SPARC, also known as osteonectin, encoded by the *SPARC* gene) upregulates TGF-β1 expression in HRMCs [49]. In our system, TGF-β1 substantially induced expression of these genes (Figure 2, left column, compare white and black bars). This was reflected in elevated secretion of the corresponding proteins (Figure 2, right column, white versus black bars). Apabetalone, JQ1, and MZ1 all opposed the TGF-β1-induced gene expression and protein secretion for each pro-fibrotic mediator, which was dose-dependent with apabetalone (Figure 2, black versus colored bars). Consistent downregulation of these factors with all three BETi confirm on-target effects. As a control, we show that blocking the TGF-β receptor with TGFBRi reduced or eliminated the response to TGF-β1 treatment as expected. Together, our results demonstrate multiple TGF-β1 stimulated factors critical for HRMC activation and pro-fibrotic response are simultaneously downregulated with BETi.

### 3.3. Apabetalone Downregulates Inflammatory Mediators in HRMCs

**Figure 3:**
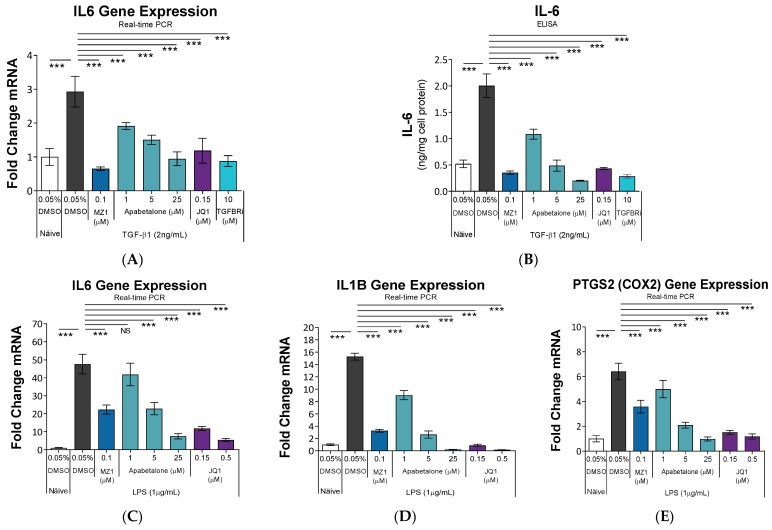
BET proteins regulate expression of inflammatory mediators. HRMCs were stimulated with TGF-β1 ± BETi or TGFBRi for 48 h (**A**,**B**) or with LPS ± BETi for 24 h (**C**–**E**). Gene expression was analyzed by real-time PCR (*n* = 4 or 5) and secreted IL-6 levels by ELISA (*n* = 3). Data are presented as mean ± SD. Statistical analysis by one-way ANOVA followed by Dunnett’s Multiple Comparison Test. *** *p* < 0.001, NS not significant. IL: interleukin. PTGS2: Prostaglandin-endoperoxide synthase 2. COX2: Cyclooxygenase 2. TGF-β1: Transforming growth factor β1. LPS: Lipopolysaccharide. Apabetalone: BD2-selective BET inhibitor. JQ1: pan-BET inhibitor. MZ1: PROTAC that directs BET proteins for degradation.

Inflammatory chemokines and cytokines produced by HRMCs incite proliferation and ECM expansion, and provoke immune cell infiltration into the mesangium [6]. IL-6 (encoded by the *IL6* gene), a cytokine of the innate immune system, is a marker of CKD-associated inflammation and directly implicated in the progression of renal dysfunction [50]. Here, we show TGF-β1 induced *IL6* gene expression and IL-6 protein secretion in HRMCs (Figure 3A,B), which was countered by BETi or TGFBRi cotreatments. Besides TGF-β1, LPS can provoke inflammatory responses leading to renal pathology, such as cytokine production in HRMC and acute kidney injury in vivo [36,51,52]. Therefore, we stimulated HRMCs with LPS, which led to robust induction of *IL6*, *IL1B*, and *PTGS2* (COX2) gene expression (Figure 3C–E). Pro-inflammatory gene expression was countered by BETi cotreatment, which was dose-dependent with apabetalone. *IL6*, *IL1B*, and *PTGS2* (COX2) are well recognized pro-inflammatory genes. Therefore, our data with LPS stimulation (Figure 3C–E) provide important evidence that apabetalone can suppress multiple inflammatory processes linked to CKD and its progression, not just those downstream of TGF-β1. Suppressed expression of inflammatory mediators with either TGF-β1 or LPS stimulation suggests apabetalone could downregulate the exaggerated inflammatory responses that accompany and perpetuate renal dysfunction.

### 3.4. Apabetalone Downregulates TGF-β1 Mediated Expression of Alkaline Phosphatase

**Figure 4:**
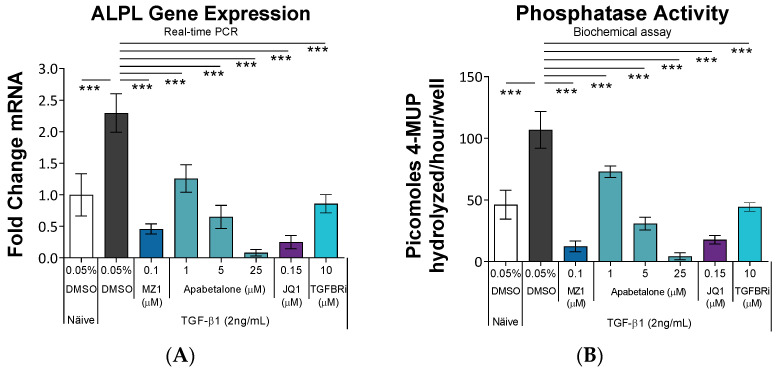
Apabetalone suppresses TGF-β1 stimulated phosphatase expression associated with calcification. HRMCs were treated with TGF-β1 ± BETi for 48 h, followed by *ALPL* gene expression analysis by real-time PCR (**A**) or cell-associated alkaline phosphatase activity using the artificial fluorescent substrate 4-methylumbelliferyl phosphate (4-MUP) (**B**). Data are presented as the mean ± SD (*n* = 5). Statistical analysis one-way ANOVA followed by Dunnett’s Multiple Comparison Test. *** *p* < 0.001. *ALPL*: Tissue-nonspecific alkaline phosphatase gene. TGF-β1: Transforming growth factor β1. Apabetalone: BD2-selective BET inhibitor. JQ1: pan-BET inhibitor. MZ1: PROTAC that directs BET proteins for degradation.

Tissue non-specific alkaline phosphatase (TNAP; encoded by the *ALPL* gene) is expressed in the kidney where it plays a beneficial role in endotoxin and nucleotide detoxification [53]. However, elevated levels of TNAP are associated with reduced glomerular function as well as soft tissue calcification [53]. We observed that *ALPL* gene expression and TNAP enzyme activity were induced by TGF-β1 (Figure 4A,B). *ALPL*/TNAP induction was suppressed at the gene-expression and consequent enzyme-activity levels by BETi. 

### 3.5. Apabetalone Alters Transcriptional Response of HRMCs to TGF-β1

**Figure 5:**
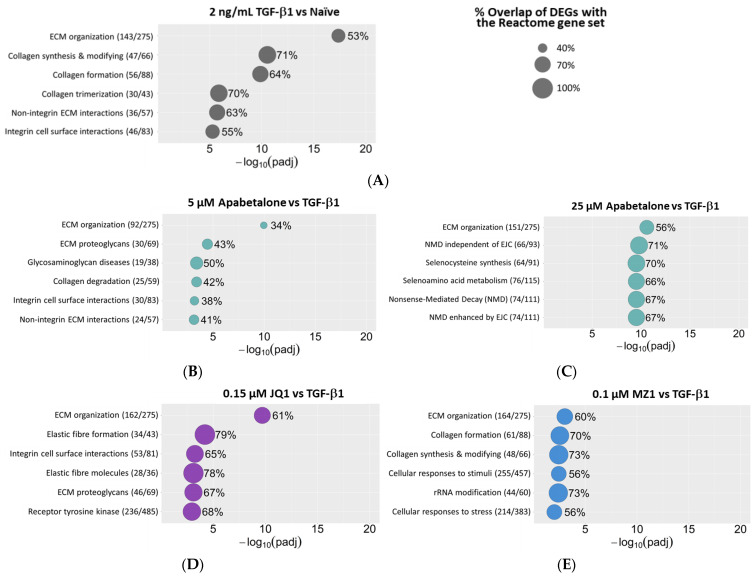
Transcriptomics show BET inhibitors affect pathways of ECM reorganization. HRMCs were treated with TGF-β1 alone (**A**) or cotreated with TGF-β1 and the indicated BETi (**B**–**E**) for 24 h, followed by transcriptomic assessment by RNA-seq. Reactome Enrichment analysis of differentially expressed genes (DEGs) identified gene sets affected by treatments. Shown are the top six most significantly enriched gene sets for each treatment. The name of the gene set is followed by the number of DEGs from RNA-seq/number of genes in that Reactome gene set (gene ratio). The size of each circle is proportional to the percent overlap between DEGs and the Reactome gene set. Statistical significance was determined using the Mann–Whitney U Test and shown as −log10(padj). TGF-β1: Transforming growth factor β1. Apabetalone: BD2-selective BET inhibitor. JQ1: pan-BET inhibitor. MZ1: PROTAC that directs BET proteins for degradation.

To further address the role of BET proteins in HRMC activation, transcriptomic profiling by RNA-seq was performed using RNA from HRMCs stimulated with TGF-β1 for 24 h in the absence or presence of BETi. Principal component analysis of the RNA-seq data showed segregation and distinct grouping of the treatment groups, indicating differential transcriptional outcomes between treatments, with modest clustering in data from 25 µM apabetalone and 0.15 µM JQ1-treated cells (Supplementary Figure S2A). Volcano plots depict the number of differentially expressed genes (DEGs) in each treatment group (adjusted *p*-value *<* 0.05; Supplementary Figure S2B). TGF-β1 treatment resulted in 4064 DEGs, with a similar number of genes downregulated (2058 genes) as upregulated (2007 genes). In contrast, all of the BETi treatments downregulated more transcripts than they upregulated, consistent with the role of BET proteins in transcriptional complexes [54]. Reactome Enrichment analysis was used to identify biological processes affected by TGF-β1 alone or with BETi cotreatment. Reactome organizes sets of genes with biological interactions into pathways, providing insight into the impact of treatments on disease processes. In cells treated with TGF-β1, the top six Reactome gene sets were related to ECM, collagen, or integrins, consistent with pro-fibrotic activation of HRMCs with profound ECM reorganization (Figure 5A). With BETi cotreatments, gene sets associated with ECM and collagen were also in the top six most affected (Figure 5B–E), indicating BET protein involvement in ECM gene expression under pro-fibrotic conditions (in Figure 5, the size of a circle is proportional to the percent of genes impacted by treatment within each gene set). “Integrin Cell Surface Interactions” appeared with TGF-β1 stimulation, and for both 5 µM apabetalone and JQ1 treatments. Intriguingly, key cell-ECM interactions that regulate fibrosis are mediated by members of the integrin family of cell adhesion molecules [55]. “ECM Organization” was the most significantly affected gene set in all treatment groups; however, direction of change (activation or repression) is not predicted by Reactome Enrichment analysis. Therefore, we examined the direction of change that each treatment imposed on DEGs in the “ECM Organization” gene set, which is composed of 275 genes in total. The complete list of DEGs contributing to the Reactome ECM Organization gene set and the effects of BETi on their expression (up- or downregulation) is shown in Supplementary Table S2 and summarized in Table 1. TGF-β1 alone resulted in downregulation of 41% of DEGs in the “ECM Organization” gene set, and upregulation of 59%, consistent with activation. BETi cotreatment with TGF-β1 resulted in downregulation of 64–69% of genes, consistent with inhibition. The “ECM Organization” gene set includes *THBS1*, *FN1*, and *SPARC*, which are key drivers of HRMC fibrosis, as highlighted in Figure 2, where BETi suppressed TGF-β1-induced expression of these genes and their corresponding proteins. The “ECM Organization” gene set also includes numerous collagen genes. Of the differentially expressed collagen genes in this gene set with TGF-β1 treatment, 70% were upregulated; however, only 13–32% were upregulated with BETi cotreatments (Supplementary Table S2). This indicates BETi leads to an overall reduction in TGF-β1 stimulated collagen production, and is congruent with reduced collagen staining observed in HRMCs cotreated with TGF-β1 and BETi (Figure 1E). Together, the Reactome Enrichment analysis indicates BETi disrupts TGF-β1-stimulated fibrotic processes associated with the ECM and its interaction with integrins.

### 3.6. Apabetalone Reduces Levels of Pro-Fibrotic Factors in Human Plasma

**Figure 6:**
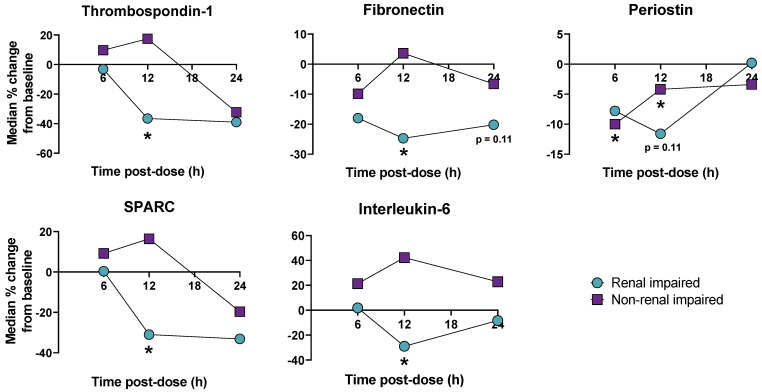
The change in plasma levels of pro-fibrotic and pro-inflammatory proteins after a single 100 mg dose of apabetalone relative to pre-dose amounts. Subjects with renal impairment had stage 4 or 5 CKD, not on dialysis. Subjects without renal impairment were matched for age, weight, and sex (eight subjects in each cohort). The Wilcoxon signed-rank test was used to evaluate changes in protein levels between pre- and post-dose levels. * *p* < 0.1. Select data have been published by Wasiak et al. [31]. h: hours.

To examine if the apabetalone effects observed in vitro were consistent with in vivo effects in humans, we assessed plasma levels of pro-fibrotic and pro-inflammatory markers in CKD patients vs. controls. We assessed eight subjects with CKD (stage 4 or 5 CKD, not on dialysis, with an eGFR of <30 mL/min per 1.73 m^2^ and a mean eGFR of 20 mL/min per 1.73 m^2^) and eight controls matched in age, weight, and sex without renal dysfunction (eGFR ≥ 60 mL/min per 1.73 m^2^, with mean eGFR of 78.5 mL/min per 1.73 m^2^). All subjects were administered a single 100 mg dose of apabetalone followed by plasma proteomics pre-dose then 6, 12, and 24 h post-dose as described by Wasiak et al. [31]. Pre-dose levels of thrombospondin-1, fibronectin, periostin, and IL-6 showed a trend to elevation in the CKD cohort for all the factors examined except SPARC, which may be consistent with the disease state promoting overproduction of these proteins (Supplementary Table S3). After apabetalone treatment, plasma levels of thrombospondin-1, fibronectin, SPARC, and IL-6 were reduced only in subjects with CKD (Figure 6). Only periostin was reduced in subjects without renal impairment who received apabetalone, while the other factors had no statistically significant change from baseline in control subjects. It is possible that downregulation of these proteins by apabetalone was more profound in patients with renal impairment because BET proteins are involved in expression of these genes in the disease state, such as those associated with TGF-β1 stimulation. 

To examine TGF-β1 signaling in CKD versus control subjects, and the impact of apabetalone on TGF-β1 transcriptional targets, we used the upstream regulator analysis in Ingenuity Pathway Analysis (IPA) software released July 2021 to interpret plasma proteomics data. If protein abundance of TGF-β1 transcriptional targets is consistently elevated or reduced in the proteome, then prediction is made about TGF-β1’s activation state (i.e., activated with z-score > 2, or inhibited with z-score < −2). Before receiving apabetalone, IPA-predicted TGF-β1 was activated in CKD subjects versus controls (Table 2, z-score 2.01, *p* < 0.05), consistent with elevated TGF-β1 signaling that accompanies kidney dysfunction [7,9,10]. After receiving apabetalone, alterations in the proteome led to a predicted inhibition of TGF-β1 in CKD subjects by 12 h and maintained at 24 h post-dose; however, no prediction was made in control subjects. Results indicate apabetalone suppressed transcriptional targets of TGF-β1 only in the human disease state, where TGF-β1 signaling is dysregulated. 

## 4. Discussion

Fibrosis and inflammation in glomeruli contribute to the pathophysiology of CKD. HRMCs are specialized cells in the central stalk of glomeruli that provide structural support for glomerular capillaries to enhance filtration [4]. In response to toxic insults or metabolic stress, HRMCs become activated to a fibrotic, contractile phenotype with increased production of ECM, inflammatory cytokines, and enzymes, all of which contribute in part to renal damage, and ultimately, dysfunction of the kidney [56]. TGF-β1 is the key fibrogenic cytokine in renal glomerular fibrosis [57]. Studies have shown that TGF-β1, mediated via its canonical SMAD signaling pathway, drives progression of renal fibrosis [58]. However, therapeutic targeting of TGF-β1 or its receptors did not slow progression of nephropathy in clinical trials [12], and also failed to reduce pulmonary fibrosis in a mouse model, while exacerbating inflammation associated with disease progression [11]. Consistent with chemical inhibition, mice with a conditional genetic knockout of TGF-β receptor II in renal tubular cells also had markedly increased renal inflammation [13]. Thus, clinical trials and pre-clinical models have demonstrated that TGF-β1 or its receptors are not suitable therapeutic strategies due to side effects and lack of efficacy. However, multiple pathways downstream of TGF-β1 have therapeutic potential to reduce HRMC activation and are regulated by BET proteins [14,21,22,23,24]. Our study demonstrates that apabetalone simultaneously suppresses pro-fibrotic and inflammatory processes induced by TGF-β1. Further, we show apabetalone reduces plasma levels of key pro-fibrotic and inflammatory markers in subjects with CKD, which is consistent with our in vitro findings [32].

Elevated α-SMA production accompanies activation of HRMCs, where it localizes to actin stress fibers and facilitates cellular contraction via interactions with integrins in focal adhesion complexes [56]. In our study, apabetalone suppressed TGF-β1-induced *ACTA2* gene expression up to 89% (*p* < 0.001 at 25 µM), and abolished α-SMA relocalization to stress fibers (Figure 1A–C), resulting in reduction in TGF-β1-stimulated cellular contraction, with complete reversal of the adverse contractile phenotype at 25 µM (*p* < 0.001; Figure 1D). Diminished staining of collagen in TGF-β1-stimulated HRMCs confirmed that apabetalone opposed ECM overproduction (Figure 1E). Apabetalone also suppressed TGF-β1-mediated induction of several key drivers of ECM overproduction and fibrosis, including thrombospondin, fibronectin, periostin, SPARC (also known as osteonectin), and interleukin 6 (Figure 2 and Figure 3A,B). Prior studies have shown each of these, on their own, are effective therapeutic targets that alleviate kidney dysfunction in pre-clinical models. For example, in inflammatory or fibrotic kidney disease, THBS1 promoted inappropriate or exaggerated TGF-β1 activation [59,60]. Inhibition with THBS1 antisense oligonucleotides or peptides blocking THBS1-TGF-β1 interactions markedly suppressed renal ECM expansion and nephritis in rats [46,47]. Accordingly, THBS1 antagonism has been recommended to treat CKD by several groups [46,61,62]. Pharmacologic inhibition of fibronectin incorporation into the ECM alleviated ischemia-reperfusion-induced kidney injury in pre-clinical models [63]. Further, inhibiting fibronectin polymerization into the ECM attenuated fibrosis and improved cardiac function in rodent and cell culture models of heart failure, another fibrosis-based disorder [64]. Periostin is not detected in the adult kidney, but is aberrantly expressed in several forms of CKD where circulating levels correlate with both the degree of fibrosis and decline in renal function [65,66]. Therefore, periostin in urine or kidney biopsy is a potential biomarker for CKD progression. In rodent models, therapeutics targeting periostin alone provided protection from chemically induced renal injury, diabetic nephropathy, or renal fibrosis, demonstrating an active role for periostin in kidney pathology [67,68]. SPARC (osteonectin) is a matricellular protein that binds collagen and is overexpressed in fibrotic diseases. Recombinant SPARC promoted TGF-β1 expression and ECM expansion by mesangial cells, implicating dysregulated SPARC expression as a driver of glomerular fibrosis [49]. In vivo, streptozotocin-induced diabetic nephropathy was less severe in SPARC-null compared to wild type mice, again demonstrating an active role for SPARC in driving kidney dysfunction [69]. Our study shows apabetalone simultaneously downregulates these validated therapeutic targets in HRMCs.

Chronic inflammation accompanies CKD, and sustained inflammatory processes evoke renal injury and exacerbate CKD progression [50]. The pro-inflammatory cytokine IL-6 is produced by activated HRMCs and linked to immune cell responses that perpetuate dysfunction in glomeruli. A neutralizing monoclonal antibody to the IL-6 receptor preserved kidney function and structure of glomeruli in a mouse model of nephritis, indicating therapeutic potential for targeting IL-6 signaling alone in CKD [70]. In patients, circulating IL-6 levels are positively associated with the severity of CKD, linking sustained IL-6 signaling with exacerbated renal dysfunction [6]. Apabetalone downregulates IL-6 expression in several cell types, including LPS-stimulated human macrophages [29], endothelial cells under a variety of inflammatory stimuli [16,26], and arterial expression in a murine model of atherosclerosis [29]. Here we extend these findings by showing apabetalone countered induction of *IL6* gene expression and secretion of IL-6 protein in TGF-β1-stimulated HRMCs, and *IL6*, *IL1B*, and *PTGS2* gene expression with LPS (Figure 3). Together, the anti-inflammatory properties of apabetalone in HRMCs, immune cells, and the vasculature may suppress inflammatory processes associated with CKD progression. 

TNAP is the most abundant isoform of alkaline phosphatase, comprising >90% of alkaline phosphatase in circulation [53]. Circulating TNAP is an independent predictor for declining renal function in patients with heart failure, emphasizing its role in kidney–heart crosstalk [71]. In clinical trials, apabetalone reduced circulating TNAP, which correlated with reductions in MACE [72]. Further, reductions in circulating TNAP were most profound in the subpopulation with CKD [32]. In mice, TNAP knockdown improved post-MI cardiac function and reduced fibrosis, while TNAP overexpression worsened cardiac function, validating TNAP as a therapeutic target for fibrotic disease [73]. In addition, TNAP promotes vascular and soft tissue calcification [74], a process that was inhibited by apabetalone in vascular smooth muscle cells [15]. Data in this study showed apabetalone downregulated TNAP expression in HRMCs (Figure 4), which may suppress fibrosis and calcification in the mesangium.

Our bioinformatic analysis of transcriptomics following HRMC treatments with TGF-β1 alone or in combination with BETi showed apabetalone impacts gene sets of ECM reorganization and integrins, suggesting apabetalone interrupts TGF-β1 stimulated fibrotic processes (Figure 5). Apabetalone disrupting gene sets involving integrins is consistent with blocking pro-fibrotic processes in HRMCs. Focal adhesion complexes, formed partly through integrin clustering, connect the actin cytoskeleton to the ECM, and are intimately involved in governing initiation, maintenance, and resolution of tissue fibrosis [75]. Integrin-containing complexes bind ECM ligands including collagen and fibronectin, which were also downregulated by apabetalone (Figure 1E, Figure 2C,D, and Supplementary Table S2). Indeed, BET proteins are known to operate at the chromatin level to drive expression of select genes dependent on cell type and disease state [54]. Epigenetic modifications to chromatin provide a mechanism for the rapid alterations in gene expression required for response to cytokine stimulation. Our data are consistent with BET protein involvement in coordinating HRMCs’ activation through epigenetic control of pro-fibrotic transcriptional programs. Thus, BETi treatment simultaneously downregulates multiple validated therapeutic targets under TGF-β1-stimulated conditions to reduce hallmark signatures of HRMC activation, such as ECM overproduction, over-enhanced contractility, and cytokine secretion. Because HRMC activation is a common pathological outcome in CKD regardless of how it is initiated, our data suggest apabetalone could have cumulative benefits in the mesangium under conditions leading to renal impairment.

Cardiovascular disease is the leading cause of death in patients with CKD [53]. Changes in plasma protein composition are a central feature of CKD pathophysiology and associated cardiovascular disease. Changes in plasma composition underlie the crosstalk between the kidney and heart, referred to as the kidney–heart axis. CKD is characterized by a strong fibrotic and inflammatory component that contributes to accelerated endothelial dysfunction, vascular inflammation, atherosclerosis, and calcification [76,77]. Current data indicate dysregulation of these processes leading to CKD-associated cardiovascular risk, in part, involve BET proteins and are modifiable with a BETi [31]. Here, we show that a single dose of apabetalone lowered plasma levels of pro-fibrotic and pro-inflammatory factors specifically in subjects with CKD (Figure 6). Further, transcriptional targets of TGF-β1 were elevated in plasma of subjects with CKD compared to controls, indicating TGF-β1 signaling was activated in CKD subjects (Table 2). Apabetalone reduced levels of transcriptional targets of TGF-β1 specifically in plasma of CKD subjects, suggesting apabetalone can suppress pro-fibrotic transcriptional signatures induced by TGF-β1 in human subjects. Although modifications in the plasma proteome are not specific to therapeutic effects in the kidney, reduced circulating levels of these proteins that operate along the kidney–heart axis are consistent with reduced risk of MACE observed in CKD patients receiving apabetalone in the phase 3 BETonMACE trial (risk of MACE was reduced by 50%, in the subpopulation with CKD defined as eGFR < 60 mL/min/1.73 m^2^, HR 0.50 95% CI 0.26, 0.96 *p* = 0.04) [32]. In vitro and in vivo human data demonstrate that selective BD2 inhibition by apabetalone has utility in reducing cardiovascular risk in CKD patients.

## 5. Conclusions

Through regulation of transcription in HRMCs, apabetalone suppressed TGF-β1-stimulated activation of HRMCs to fibroblast-like cells associated with nephropathy. Fibrotic and inflammatory factors suppressed by apabetalone in cultured HRMCs were also reduced in the plasma of CKD patients receiving apabetalone, with substantially less impact in matched subjects without renal dysfunction. Results indicate BET proteins are involved in dysregulated processes that accompany CKD regardless of how CKD is initiated, and may be modified through BET inhibition with apabetalone, a clinical-stage therapeutic. Through crosstalk along the kidney–heart axis, effects of apabetalone on fibrotic, inflammatory, and calcific processes in the kidney may reduce cardiovascular risk.

## Figures and Tables

**Table 1:** The effect of BETi on expression of genes in the “ECM Organization” gene set from the Reactome database. ECM: extracellular matrix. DEGs: differentially expressed genes. TGF-β1: Transforming growth factor β1. Apa: apabetalone, a BD2-selective BET inhibitor. JQ1: pan-BET inhibitor. MZ1: PROTAC that directs BET proteins for degradation.

	2 ng/mL TGF-β1				
	0.05% DMSO	5 µM Apa	25 µM Apa	0.15 µM JQ1	0.1 µM MZ1
# DEGs in “ECM Organization” Gene Set	143	92	151	162	164
# Genes Upregulated	84	33	47	52	54
% Upregulated	59%	36%	31%	32%	33%
# Genes Downregulated	59	59	104	110	110
% Downregulated	41%	64%	69%	68%	67%

**Table 2:** IPA upstream regulator analysis of TGF-β1 via plasma proteomics. Plasma levels of 1305 proteins were determined in subjects with renal impairment (CKD; *n* = 8) and controls without renal impairment matched for age, weight, and sex (*n* = 8) following a single dose of apabetalone (100 mg). Orange indicates predicted activation (IPA z-score > 2, and Benjamini–Hochberg adjusted *p* < 0.05), while blue indicates predicted inhibition (IPA z-score < −2, *p* < 0.05). TGF-β1: Transforming growth factor β1. Apabetalone: BD2-selective BET inhibitor.

TGF-β1 IPA Upstream Regulator Analysis					
Pre-dose:			Time post-apabetalone dose		
			6 h	12 h	24 h
CKD vs. Controls	2.01 Activated	CKD vs. pre-dose	1.15	−2.66	−3.49
			No prediction	Inhibited	Inhibited
		Controls vs. pre-dose	−0.35	0.88	−0.44
			No prediction	No prediction	No prediction

## Data Availability

Data presented in this study are available upon reasonable request from the corresponding author.

## Supplementary Materials

The following supporting information can be downloaded at: https://www.mdpi.com/article/10.3390/biomedicines11061663/s1, Table S1: TaqMan Assay IDs used in this study, Figure S1: Assessment of toxicity of treatments to HRMCs, Figure S2: TGF-β1 stimulation with and without BETi cotreatments result in unique transcriptional signatures, Table S2: The effect of BETi on expression of genes of the “ECM Organization” gene set from the Reactome database, Table S3: Pre-dose levels of plasma proteins.
