# Supplementary material for: Apabetalone Downregulates Fibrotic, Inflammatory and Calcific Processes in Renal Mesangial Cells and Patients with Renal Impairment
Source: Biomedicines. 2023 Jun 8;11(6):1663. doi: 10.3390/biomedicines11061663 (PMC10295623; doi:10.3390/biomedicines11061663)
Supplement: Supplementary file 1 [file biomedicines-11-01663-s001.zip › biomedicines-2416657-supplementary/Suppl Table S1 TaqMan IDs.pdf]

Gilham et al. Apabetalone Downregulates Fibrotic, Inflammatory and Calcific Processes in Renal Mesangial Cells and Patients with Renal Impairment

Supplemental Table S1: TaqMan Assay IDs used in this study (obtained from ThermoFisher Scientific)

Gene	Protein	TaqMan Assay ID
ACTA2	Alpha smooth muscle actin	Hs05005341_m1
THBS1	Thrombospondin 1	Hs00962908_m1
FN1	Fibronectin	Hs01549976_m1
POSTN	Periostin	Hs01566750_m1
SPARC	SPARC or Osteonectin	Hs00277762_m1
IL6	Interleukin-6	Hs00174131_m1
IL1B	Interleukin-1 beta	Hs00174097_m1
PTGS2	Cyclooxygenase 2	Hs00153133_m1
ALPL	Tissue non-specific alkaline phosphatase	Hs01029144_m1
PPIA	Cyclophilin A	4326316E
