# Supplementary material for: Apabetalone Downregulates Fibrotic, Inflammatory and Calcific Processes in Renal Mesangial Cells and Patients with Renal Impairment
Source: Biomedicines. 2023 Jun 8;11(6):1663. doi: 10.3390/biomedicines11061663 (PMC10295623; doi:10.3390/biomedicines11061663)
Supplement: Supplementary file 1 [file biomedicines-11-01663-s001.zip › biomedicines-2416657-supplementary/Suppl Table S3 Pre-Dose Levels of Plasma Proteins.pdf]

Gilham et al. Apabetalone Downregulates Fibrotic, Inflammatory and Calcific Processes in Renal Mesangial Cells and Patients with Renal Impairment

Supplemental Table S3: Baseline (pre-dose) plasma levels of pro-fibrotic and pro-inflammatory proteins. Subjects with renal impairment had stage 4 or 5 CKD, not on dialysis. Subjects without renal impairment were matched for age, weight, and sex (8 subjects in each cohort). Data are shown as relative fluorescence units (RFU) determined by SOMAscan. Statistical analysis between cohorts by Mann-Whitney U-test. Bold indicates $p < 0.1$.

Baseline plasma levels (mean RFU ± SEM)			
	**Renal impaired**	**Non-renal impaired**	***p* value**
Thrombospondin-1	6,899 ± 1,285	6,017 ± 691	0.645
Fibronectin	6,882 ± 2,475	5,181 ± 1,129	0.879
Periostin	5,753 ± 435	4,419 ± 200	**0.015**
SPARC (osteonectin)	41,172 ± 5,015	43,201 ± 5,204	0.798
Interleukin-6	776 ± 138	518 ± 57	0.105
