# Supplementary material for: Apabetalone Downregulates Fibrotic, Inflammatory and Calcific Processes in Renal Mesangial Cells and Patients with Renal Impairment
Source: Biomedicines. 2023 Jun 8;11(6):1663. doi: 10.3390/biomedicines11061663 (PMC10295623; doi:10.3390/biomedicines11061663)
Supplement: Supplementary file 1 [file biomedicines-11-01663-s001.zip › biomedicines-2416657-supplementary/Supplemental Figures.pdf]

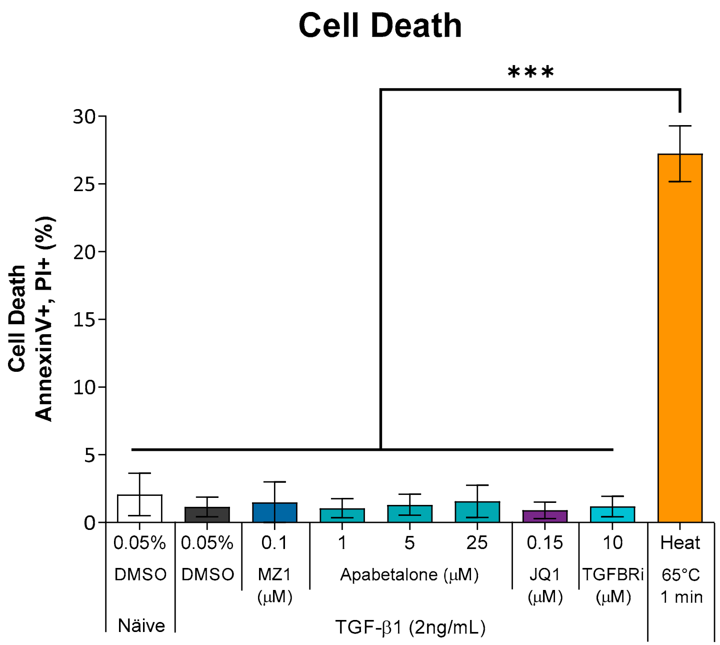

Supplemental Figure S1: Assessment of toxicity of treatments to HRMCs. HRMC were treated for 24 h followed by analysis of toxicity by annexin V and propidium iodide staining using flow cytometry. One group was exposed to 65°C for 1 minute to induce cell death. Data represent the mean ± standard deviation of 3 independent experiments. Statistical analysis by one-way ANOVA followed by Tukey's Multiple Comparison Test. ***p<0.001 between groups. TGF-β1: Transforming growth factor β1. TGFBRi: small molecule inhibitor of the TGF-β receptor. Apabetalone: BD2-selective BET inhibitor. JQ1: pan-BET inhibitor. MZ1: PROTAC that directs BET proteins for degradation. PI: propidium iodide.

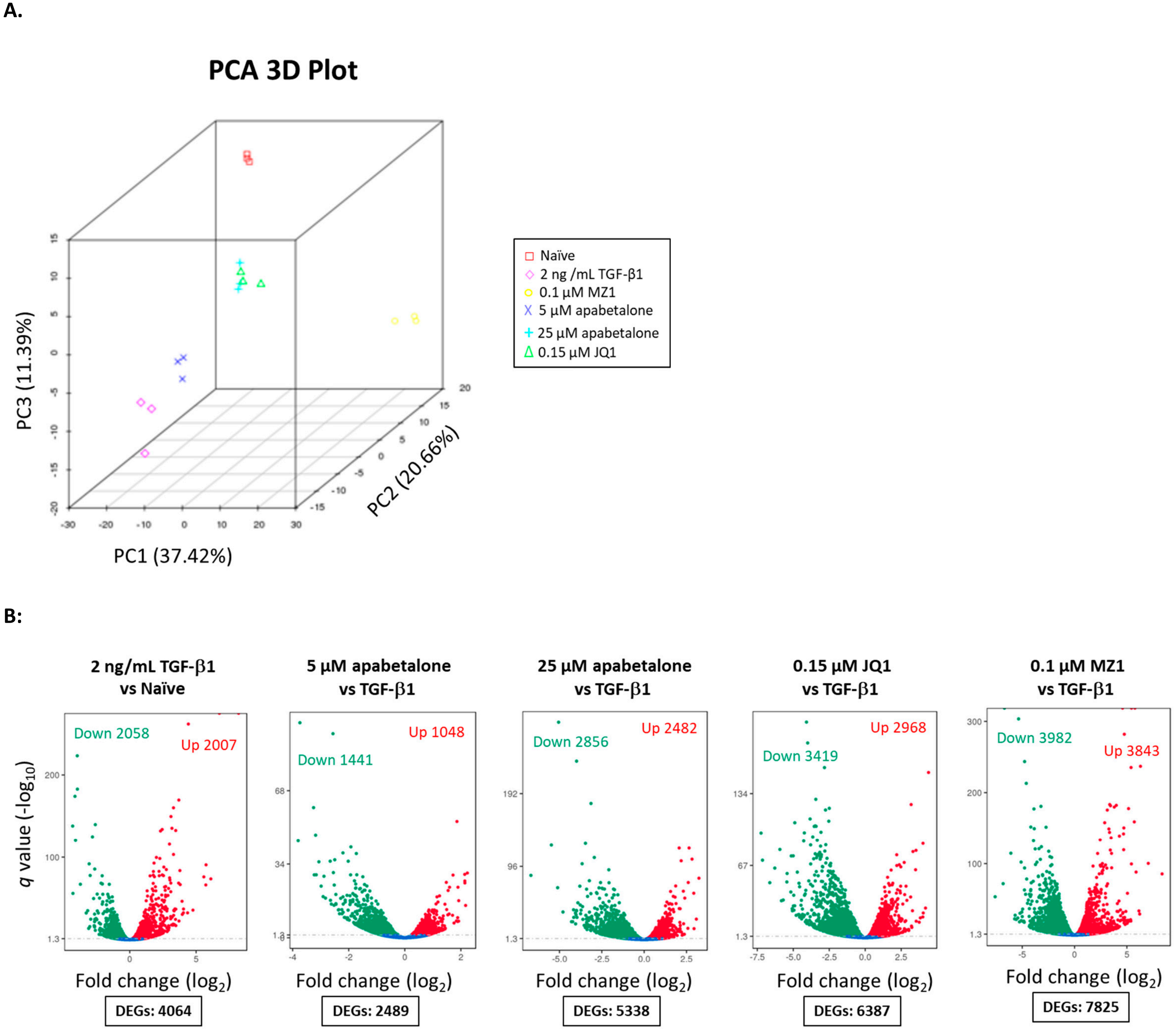

Supplemental Figure S2: TGF-β1 stimulation with and without BETi cotreatments result in unique transcriptional signatures **(A)** Principal component analysis (PCA) for Naïve, TGF-β1 stimulated, and TGF-β1 + BETi treated samples. PCA was completed by DESeq2 R package and

shows differentiation between treatment groups. **(B)** Volcano plots of differential gene expression (DEG; significance cutoff: *p*adj < 0.05). Horizontal axis indicates the magnitude of change in gene expression. Vertical axis indicates the Benjamini-Hochberg adjusted p-values. Each dot represents a gene; blue dots indicate no significant difference in expression; red dots indicate upregulated genes; green dots indicate downregulated genes. TGF-β1: Transforming growth factor β1. Apabetalone: BD2-selective BET inhibitor. JQ1: pan-BET inhibitor. MZ1: PROTAC that directs BET proteins for degradation. DEGs: differentially expressed genes.
